# Arrhythmogenic Right Ventricular Cardiomyopathy/Dysplasia^1^

**Published:** 2003-07-01

**Authors:** Julia H Indik, Frank I Marcus

**Affiliations:** Sarver Heart Center, University of Arizona, Tucson AZ

## Abstract

Arrhythmogenic right ventricular cardiomyopathy/dysplasia (ARVC/D) is characterized by the patchy replacement of myocardium by fatty or fibrofatty tissue. These changes lead to structural abnormalities including right ventricular enlargement and wall motion abnormalities that can be detected by echocardiography, angiography, and cine MRI. ARVC/D is a genetically heterogeneous disorder, since it has been linked to several chromosomal loci. Myocarditis may also be a contributing etiological factor. Patients are typically diagnosed during adolescence or young adulthood. Presenting symptoms are generally related to ventricular arrhythmias. Concern for the risk of sudden cardiac death may lead to the implantation of an intracardiac defibrillator. An ongoing multicenter international registry should further our understanding of this disease.

## Introduction

Arhythmogenic right ventricular cardiomypathy/dysplasia (ARVC/D) is a disorder in which the right ventricular myocardium is interspersed with fatty or fibrofatty tissue. This pathological process principally affects the right ventricle, but in advanced cases can lead to biventricular involvement. Linkage analysis has identified several loci on different chromosomes, indicating this disease is genetically heterogeneous. Specific mutations have been identified in the genes for desmoplakin [1], plakoglobin [2] and the ryanodine receptor gene [3]. The predominant presenting symptoms are due to ventricular arrhythmias, including palpitations, sustained ventricular tachycardia, or uncommonly, sudden cardiac death. Although ARVC/D is an unusual disorder, it has been described as the most common cause of sudden death in young athletes under the age of 35 in the Veneto area in Italy [4] . Heart failure is infrequent but may occur due to severe right or biventricular enlargement.

### a) Structural Abnormalities and Pathophysiology

**Figure 1:**
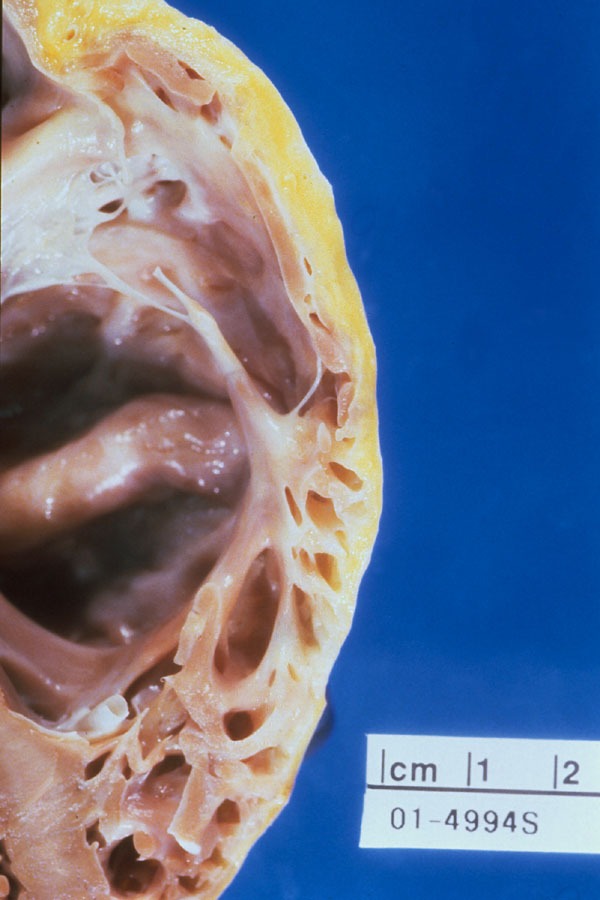
In ARVC/D there is progressive fibro-fatty replacement of the myocardium with thinning and enlargement of the RV wall. In this specimen there is gross fatty infiltration seen in a portion of the RV wall.

**Figure 2:**
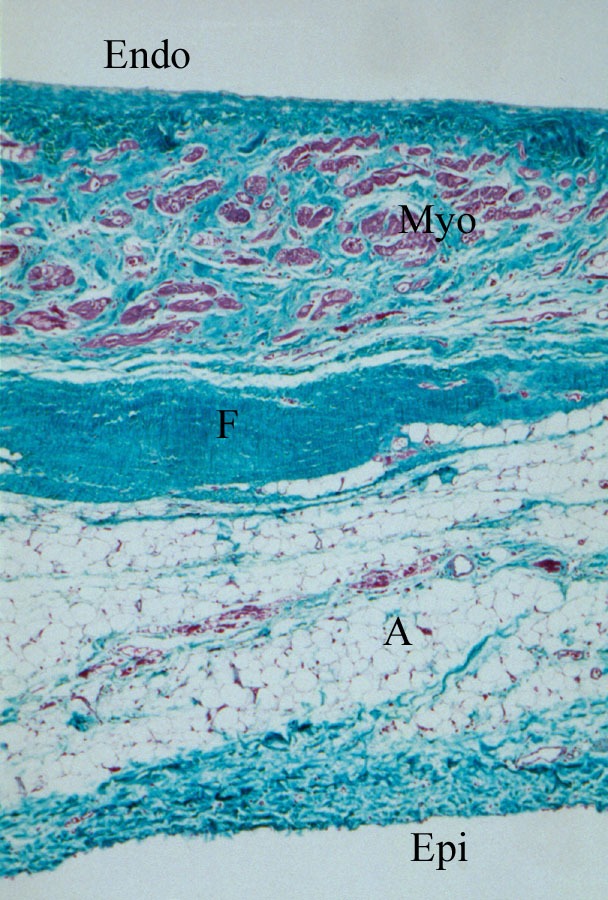
Trichrome staining shows areas of mature fibrosis (F) and adipose tissue (A) within the epicardial (Epi) and mid-myocardial zones, with remaining small clusters of myocytes (Myo) near the endocardial edge (Endo).

Anatomically, ARVC/D is characterized by replacement of myocardium with fatty and fibrous tissue that primarily affects the epicardium and the mid-myocardium, with relative sparing of the endocardium (Figure 1 and 2). This process most commonly affects the posterior and inferior areas of the right ventricular inflow tract adjacent to the tricuspid valve [5], but it also affects the anterior infundibulum and the apex, thus forming what is known as the "triangle of dysplasia" [6]. Loss of myocardium may result in aneurysm formation, commonly in the basal inferior wall, underneath the tricuspid valve [7]. Fibro-fatty replacement occurs in a segmental, patchy fashion and adjacent areas, particularly, the septum, are generally spared. Therefore, endomyocardial biopsies, which usually are performed from septal tissue, may be non-diagnostic. ARVC/D is distinguished from Uhls anomaly, a congenital disorder in which right ventricular myocardium is absent, resulting in a paper-thin right ventricular wall [8,9]. Recently, apoptotic cells have been reported in endomyocardial biopsy specimens [10-13]. It is unknown how the apoptotic pathway may be initiated in ARVC/D, but may relate to altered intracellular calcium concentration [1]. Inflammatory infiltrates consisting of lymphocytes may also be found on biopsy specimens, suggestive of a focal myocarditis, and may explain sporadic cases of ARVD/C.

Diffuse hypokinesis or regional wall motion abnormalities of the right ventricle can be seen with contrast ventriculography, considered to be the most reliable test to assess structural abnormalities. RV angiography should be performed in four views: RAO 30, LAO 60, AP and lateral. Affected areas will show hypokinesis, akinesis, or dyskinesis. Wall motion abnormalities can also be observed by echocardiography but particular attention must be focussed on imaging the basal posterior and inferior walls near the tricuspid valve [5,14]. MRI is emerging as a useful non-invasive tool to identify morphologic abnormalities such as myocardial fat, right ventricular chamber size and shape, and wall motion abnormalities with newer cine techniques. However, with conventional MR technology, the identification of myocardial fat was not found to be a reliable predictor of the presence of ARVC/D [15].

### b) Electrocardiographic Abnormalities

**Figure 3:**
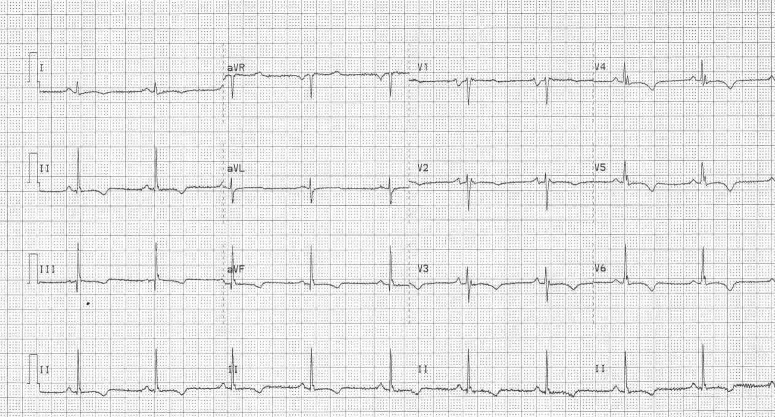
This ECG from a patient with ARVC/D shows T wave inversion throughout the precordial leads.

**Figure 4:**
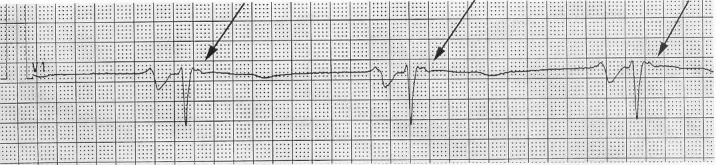
Epsilon waves, representing right ventricular late potentials, can be brought out by recording the ECG at double speed (50mm/s), double amplitude (20mm/mV), and a 40 Hz filter. Here, epsilon waves are evident as small, notched deflections just after the QRS in lead V1 (arrows).

Electrocardiographic changes include inverted T waves in the right precordial leads beyond V1 (Figure 3) in the absence of right bundle branch block [16,17]. Right ventricular late potentials in the form of epsilon waves may be found on the routine 12 lead ECG. If not present overtly, they may be identified by recording the ECG at double standard speed (50mm/sec) and amplitude (20mm/mV) and utilizing a 40 Hz filter [5] (Figure 4) . Evidence of right ventricular parietal block is a QRS duration that is longer in the right (leads V1, V2 and V3) than in the left (V4, V5, V6) precordial leads. Suggested parameters to verify this include a localized QRS duration greater than 110ms in the right precordial leads, or a maximum QRS duration in leads V1, V2 or V3 or more than 25ms above the QRS duration in lead V6 in the presence of incomplete or complete right bundle branch block [18]. The signal averaged electrocardiogram is often abnormal and supports the diagnosis of ARVC/D, but if normal, does not exclude the diagnosis. The sensitivity of the signal averaged electrocardiogram can be improved by using a 25 Hz high pass filter and paying particular attention to the Z lead [19].

### c) Arrhythmias

The ventricular arrhythmias generally arise from the right ventricle, and therefore have a left branch block morphology. Right ventricular outflow tract tachycardia (RVOT), must be excluded as this diagnosis carries a benign prognosis and is not hereditary5 . RVOT tachycardia has a left bundle branch block morphology. The QRS points to the right and inferior (positive in leads II, III, AVF and negative in AVL); this morphology can also be seen in ventricular tachycardia due to ARVC/D. Patients with ARVC/D may also experience supraventricular arrhythmias [20] including atrial flutter [21-23].

### d) Genetics

Thirty to fifty percent of patients with ARVC/D will have evidence of familial disease [25,26]. The variable clinical expression and course is believed to be at least in part due to genetic heterogeneity. Inheritance is autosomal dominant, with the exception of Naxos disease, which is autosomal recessive. Linkage analysis in families with ARVD has revealed several loci for this disorder on chromosomes 1, 2, 3, 10, 14, and 17 [26-31] (see Table 1). The mutation for ARVD8 has been identified in a gene for desmoplakin on chromosome 6, a component of desmosomes that are cellular adhesion proteins responsible for cellular binding [1]. This is the first gene identified to cause a major form of the disease, with autosomal dominant inheritance. The locus on chromosome 1 leads to a form of ARVC/D that is notable for exertional polymorphic, instead of monomorphic, ventricular tachycardia [3,32] and has been further identified to encode a cardiac ryanodine receptor gene that is responsible for calcium release from the sarcoplasmic reticulum. This genetic mutation has also been implicated in familial catecholaminergic polymorphic ventricular tachycardia [33,34]. Naxos disease discovered on the Greek island, Naxos, is characterized by palmoplantar keratosis and woolly hair in addition to RV dysplasia. Naxos disease has been mapped to chromosome 17 involving a mutation in the gene that encodes plakoglobin, a component of desmosomes and adherens junctions responsible for maintaining tight adhesions between cells [2]. Another mutation in the gene for desmoplakin that also causes an autosomal recessive syndrome with woolly hair and a dilated left ventricle has been reported in patients from Ecuador [35]. Therefore, ARVD can result from a variety of genetic mechanisms, and in particular, appears to be a disorder of proteins involved in cellular adhesion and calcium release.

Not all cases of ARVD are inherited since mutations may occur sporadically. Nonetheless, because of the genetic basis for this disorder, it is recommended that first degree relatives of ARVD patients be screened with an ECG and echocardiogram [5]. Other non-invasive tests to identify affected family members include signal-averaged ECG, Holter monitor and exercise stress testing.

## Other Contributory Factors

A large number of ARVC/D patients are noted to be athletes, and sudden cardiac death is more likely during exertion [4]. Athletic activity, possibly through stretch of myocardial fibers or elevated catecholamines may accelerate the degeneration of the ventricular myocardium and leads to ventricular arrhythmias. Also in support of the importance of other etiologic factors are the reports of Coxsackie virus B3 biopsy specimens [36], and recently, enterovirus and adenovirus by polymerase chain reaction in 7 of 12 specimens from sporadic cases [37]. This raises the possibility that the myocardium in ARVD is particularly vulnerable to inflammatory processes. A superimposed myocarditis could result in lymphocytic infiltrates and fibrofatty replacement of the myocardium [38,39]. It is not known whether the detection of viral DNA or RNA indicates that right ventricular cardiomyopathy is a result of viral myocarditis or this cardiomyopathy predisposes the heart to viral infection. This process may only affect the RV, but can affect both ventricles as well. Patients with left ventricular involvement tend to be older and more symptomatic from both arrhythmias and heart failure, consistent with the view that the disease is progressive [40].

Since there is no single "gold standard" diagnostic test to reliably verify this condition, a combination of diagnostic criteria have been established by the Task Force of the Working Group on Myocardial and Pericardial Disease [41]. Major criteria include demonstration of severe wall motion abnormalities, fibrofatty replacement by biopsy, epsilon waves and family history of histologically confirmed disease. Minor criteria include milder alterations of ventricular function, inverted T waves in the right precordial leads, late potentials, ventricular tachycardia with left bundle branch morphology or frequent premature ventricular complexes (>1000 PVCs in a 24 hour period), or family history of suspected or clinically diagnosed ARVC/D. A diagnosis can then be made if at least two major, 1 major and 2 minor, or 4 minor criteria from different categories are fulfilled Disease [41].

A prospective study of the course of disease in 37 affected families with 365 relatives in Italy showed that 41% could be diagnosed with certainty to have ARVC/D while in another 11% the diagnosis was uncertain Disease [42]. Patients were diagnosed mostly during adolescence and early adulthood. After a mean follow-up of 8.5 years, an additional 15 subjects developed structural abnormalities or ventricular arrhythmias. In an evaluation of relatives of 67 ARVC/D patients in the United Kingdom, 28% of patients had relatives that satisfied the Task Force criteria, but another 20% had relatives with only minor ECG, echo or Holter abnormalities suggestive of early or mild disease Disease [43]. Thus familial disease may have been present in up to 48% of index cases, which is plausible for a disease with autosomal dominant inheritance. It has been suggested by these authors that the standard diagnostic criteria should be modified to identify ARVC/D in relatives of patients with confirmed disease if they satisfy any one of the following abnormalities: i) T-wave inversion in the precordial leads ii) abnormal SAECG iii) ventricular tachycardia with left bundle branch morphology during ECG, Holter monitoring or exercise testing iv) greater than 200 PVCs in a 24 hour period or v) any mild echocardiographic abnormalities such as RV dilatation with preserved LV function Disease [42] .

## Treatment/Management

Therapy with beta blockers Disease [44], sotalol [44] or amiodarone [45] may be effective in suppressing ventricular arrhythmias and possibly in preventing sudden cardiac death. Implantation of an ICD may also be indicated to prevent sudden death. Catheter ablation of ventricular tachycardia may be useful in patients with refractory symptoms despite antiarrhythmic therapy. However, ventricular arrhythmias may recur from other areas. Patients should also be advised not to perform vigorous exercise or engage in competitive sports. Surgical disarticulation of the right ventricular free wall from its attachments to the left ventricle and septum can prevent the electrical propagation of ventricular arrythmias from the right to the left ventricle [46,47]. This was an effective means to prevent sudden death prior to the availability of the ICD, but resulted in severe right ventricular failure. Management of heart failure includes standard medical therapy with consideration of heart transplantation if severe ventricular, especially biventricular, dysfunction is present.

It is important to emphasize that risk factors for sudden death are not well characterized. A history of syncope, right or left ventricular abnormalities seen on radionuclide angiography and QRS dispersion greater than 40ms are independent predictors of sudden death [48]. However, the absence of these factors cannot provide absolute assurance that a patient or relative is free of risk. The diverse phenotypic expression of ARVC/D is still being appreciated as new genetic loci are discovered. Furthermore, the risk of sudden death is probably variable among different types of ARVC/D. In Newfoundland, Canada, ARVD5 was reported to cause sudden death in 44% of affected males, while females had a more benign course with no sudden deaths. Thus, determining prognosis is complicated by the genetic heterogeneity of this disorder.

It is unclear how to advise family members of individuals that have died suddenly with ARVD. The eight-year actuarial probability of developing ventricular arrhythmias in asymptomatic family members with normal echocardiograms was found to be 3%, with no sudden deaths [50]. The potential risks associated with the implantation and long-term management of an intracardiac defibrillator in family members with suspected disease but minimal right ventricular dysfunction may far outweigh its potential benefits. Counseling of family members needs to be individualized.

The Multidisciplinary Study of Right Ventricular Dysplasia is a multicenter study funded by the National Institutes of Health and National Heart, Lung, and Blood Institutes that is enrolling patients with a recent diagnosis of ARVC/D into a North American registry. A large registry of patients is needed to answer important questions related to diagnosis of the disorder, in particular in family members, as well as clinical management of affected patients and risk stratification for sudden death. This study will also facilitate genetic research to identify other specific mutations and their role in the pathogenesis of this disease [51,52].

## Figures and Tables

**Table 1:**
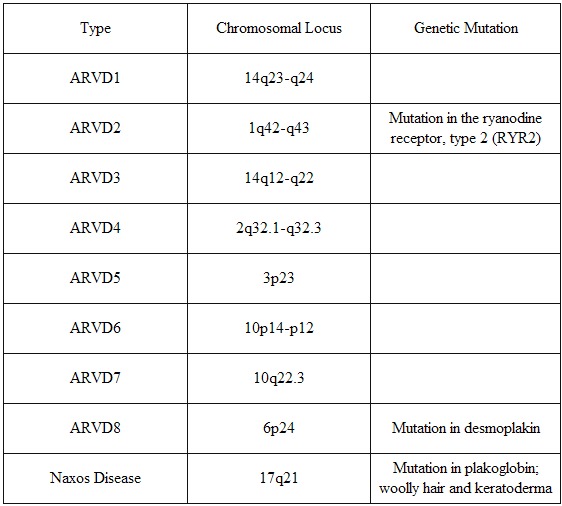
ARVC/D Types

ARVD types 1 through 8 are inherited as an autosomal dominant condition, while in Naxos disease the inheritance is autosomal recessive.
